# The Yellow-browed Warbler (*Phylloscopus inornatus*) as a model to understand vagrancy and its potential for the evolution of new migration routes

**DOI:** 10.1186/s40462-022-00345-2

**Published:** 2022-12-14

**Authors:** Paul Dufour, Susanne Åkesson, Magnus Hellström, Chris Hewson, Sander Lagerveld, Lucy Mitchell, Nikita Chernetsov, Heiko Schmaljohann, Pierre-André Crochet

**Affiliations:** 1 LECA, CNRS, Univ. Grenoble Alpes, Univ. Savoie Mont Blanc, Grenoble, France; 2 Department of Biological and Environmental Sciences, University of Gothenburg, Gothenburg, Sweden; 3 Gothenburg Global Biodiversity Centre, Gothenburg, Sweden; 4 Department of Biology, Center for Animal Movement Research, Lund University, Ecology Building, 22362 Lund, Sweden; 5 Ottenby Bird Observatory, Öland, Sweden; 6 British Trust for Ornithology, The Nunnery, Thetford, Norfolk, IP27 2PU UK; 7 Wageningen University & Research, Ankerpark 27, 1781 AG Den Helder, Netherlands; 8 Environmental Research Institute, Centre for Energy and Environment (CfEE), The North Highland College UHI, Ormlie Road, Thurso, KW14 7EE UK; 9 Ornithology Lab, Zoological Institute RAS, 1 Universitetskaya Emb, 199034 St. Petersburg, Russia; 10 Department of Vertebrate Zoology, St. Petersburg State University, 7-9 Universitetskaya Emb, 199034 St. Petersburg, Russia; 11 Institute for Biology and Environmental Sciences (IBU), Car Von Ossietzky University of Oldenburg, Carl-Von-Ossietzky-Straße 9-11, 26129 Oldenburg, Germany; 12 Institute of Avian Research, An Der Vogelwarte 21, 26386 Wilhelmshaven, Germany; 13 CEFE, CNRS, Univ Montpellier, EPHE, IRD, Montpellier, France

**Keywords:** Migration route, Orientation, Seasonal migration, Songbirds, Vagrancy, Yellow-browed Warbler

## Abstract

Why and how new migration routes emerge remain fundamental questions in ecology, particularly in the context of current global changes. In its early stages, when few individuals are involved, the evolution of new migration routes can be easily confused with vagrancy, i.e. the occurrence of individuals outside their regular breeding, non-breeding or migratory distribution ranges. Yet, vagrancy can in theory generate new migration routes if vagrants survive, return to their breeding grounds and transfer their new migration route to their offspring, thus increasing a new migratory phenotype in the population. Here, we review the conceptual framework and empirical challenges of distinguishing regular migration from vagrancy in small obligate migratory passerines and explain how this can inform our understanding of migration evolution. For this purpose, we use the Yellow-browed Warbler (*Phylloscopus inornatus*) as a case study. This Siberian species normally winters in southern Asia and its recent increase in occurrence in Western Europe has become a prominent evolutionary puzzle. We first review and discuss available evidence suggesting that the species is still mostly a vagrant in Western Europe but might be establishing a new migration route initiated by vagrants. We then list possible empirical approaches to check if some individuals really undertake regular migratory movements between Western Europe and Siberia, which would make this species an ideal model for studying the links between vagrancy and the emergence of new migratory routes.

## Background

Spectacular long-distance movements of birds have fascinated mankind since ancient times. Albatrosses routinely fly several thousands of kilometres during single foraging trips (e.g. [1, 2]), whereas terns [3], shearwaters [4], waders [5] and songbirds [6–8] regularly fly across and connect different continents and oceans during seasonal migrations. In the most extreme cases, individual birds may cover distances of more than 40,000 km ([3], seasonal migration of Arctic Tern *Sterna paradisea*) or 184,000 km on average [9], postnatal dispersal of Wandering albatross *Diomedea exulans*) in a single year.

In addition to their regular movements, birds are renowned for their propensity to move away from their geographic range limits, a phenomenon called vagrancy and defined as the occurrence of individual birds outside their regular breeding grounds, non-breeding areas or migratory flyways [10, 11]. The ornithological literature is ripe with papers describing the occurrence of extreme (e.g. [12, 13]) as well as less spectacular vagrants (e.g. [14]). Although such anecdotal observations exemplify the incredible movement abilities of some bird species, their evolutionary and ecological implications have long been regarded as negligible, as vagrancy generally concerns few individuals, which usually do not survive the long-distance movements off their normal flyway and are, therefore, lost from the gene pool of the population [11, 15]. Yet, vagrancy has been suggested to promote the colonisation of new breeding ranges [16, 17] or facilitate the long-distance dispersal of other organisms [18, 19]. In addition, in species where migration is under strong genetic control (e.g. songbirds: [20, 21]), or is passed on through social learning (e.g. geese: [22]), a vagrant following a novel route that survives, returns to its breeding grounds and successfully reproduces could transmit its successful migration phenotype to the next generation. If the frequency of such atypical movements then increases in some populations, so that more individuals survive these journeys and return to the breeding area in the following years, vagrancy could then foster the emergence of novel migration routes (see [8, 23]). In this paper, we will discuss the conceptual framework and empirical challenges of distinguishing regular migration from vagrancy in small, obligate migratory passerines and illustrate how this can enlighten our understanding of the evolution of migration, using the Yellow-browed Warbler (*Phylloscopus inornatus*; YBW hereafter) as an example of a species that has recently increased significantly outside its traditional migratory flyway.

**Fig. 1:**
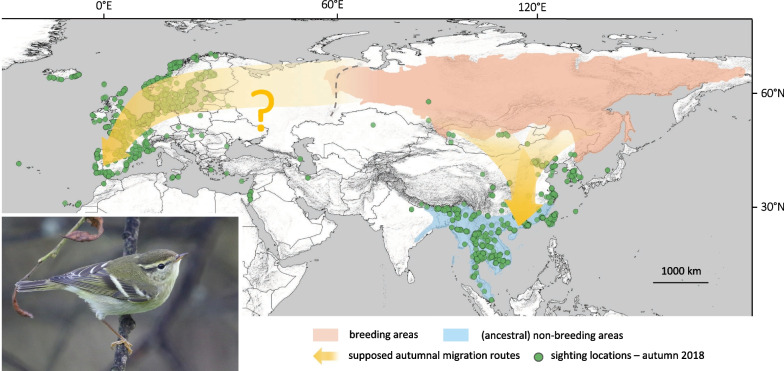
Distribution ranges and possible migration routes of the Yellow-browed Warbler (*Phylloscopus inornatus*). Migration routes in Asia (orange) indicated are based on the information collected in the literature [24]. The presumed route to Europe is hypothetical, it follows the areas where the species is most abundant during the autumn period between the most western breeding sites (Ural Mountains, indicated by a dotted line) and the areas where the species is regular in winter (i.e., Iberian Peninsula [26]). Breeding (light red) and non-breeding ranges (blue) are taken from BirdLife International [126]. Green dots represent the locations of YBW sightings between September and November 2018 provided by GBIF [127]. The majority of sightings are concentrated in Western Europe and south-east Asia with some exceptions in the Middle East and Central Asia. Note that the frequency of the green dots does not reflect the frequency of occurrence of the species in the correct proportion due to an observer/recorder bias. The photo shows a Yellow-browed Warbler observed on Ouessant Island, France, during 2020 fall (Photo Frédéric Veyrunes)

The YBW is a small (< 8 g) long-distance songbird migrant that breeds in northern Siberia from the Ural Mountains to the Pacific Ocean and winters in south-east Asia (Fig. 1, [24]). When leaving the western breeding areas, YBWs likely first head east/southeast to reach southeastern Siberia / northeastern Mongolia, where they follow then a south/south-west direction towards south-east Asia [24]. In Europe, the YBW has become a regular autumn visitor in the last 30 years along the western European flyway, with thousands of birds occurring each autumn on a large front from Scandinavia to the Iberian Peninsula each autumn (Fig. 1). Following this dramatic increase, the number of winter records and overwintering individuals of YBW has steadily risen in western and southern Europe ([25, 26], see Sect. 2 below).

Two factors may explain the recent increase of YBW numbers in Western Europe. Firstly, most of the YBWs seen in Europe outside their normal migration route might be misoriented juveniles, which migrate in the “wrong” direction during their inaugural migration, i.e., vagrants [27, 28]. Secondly, the increase in the number of sightings in Europe suggests that the species might be establishing a new migration route in a completely different direction from its ancestral wintering grounds (Fig. 1) as proposed by Gilroy and Lees [29] in the context of the general increase of Siberian bird species in Europe (their “pseudo-vagrancy” hypothesis). These factors are not mutually exclusive, so that vagrancy and migration can both contribute to the appearance of YBWs in Europe. Distinguishing between these mechanisms remains challenging because the phenomenon “vagrancy” lacks a conceptualized definition and is, therefore, difficult to separate from the phenomenon “migration” (see [30]). Moreover, it is impossible to identify whether a single bird is a migrant or vagrant if, as is likely, the bird’s migratory history and future remain unknown.

Regardless of the relative contribution of migration and vagrancy to the status of YBW in Europe, the species stands out as an ideal model to better understand the evolutionary bases of variation on migration orientation, in species with strong genetic control of the migratory phenotype (in time and space, [20, 21, 31]). If the YBW is still only a vagrant in Europe (i.e., without individuals undertaking a regular migration to Europe and back to the breeding areas), the numbers of birds involved in these movements and the annual recurrence of this phenomenon make this model a unique opportunity to study the mechanisms underlying vagrancy behaviour. It would in fact be extraordinary to see thousands of vagrants engaging in an evolutionary dead-end every autumn. Conversely, if a new migration route is currently evolving westward in the YBW, the species would join the very few documented cases of contemporary changes of migration routes in birds with genetically encoded migrations (Eurasian Blackcap *Sylvia atricapilla*: [20]; Barn Swallow *Hirundo rustica*: [32]; Cliff Swallow *Petrochelidon pyrrhonota*: [33]; possibly Richard’s Pipit *Anthus richardi*: [8]; see also [34–36] for other examples of change of migration routes) and would thus stand out as a prime model to improve our understanding of how new migration routes emerge.

In this paper, we discuss the annual occurrences of YBW in Europe in relation to the two non-mutually exclusive factors that can contribute to this phenomenon: vagrancy vs. seasonal migration. More generally, we also aim to discuss available evidence and possible future results suggesting that vagrancy might be part of the process giving rise to new migration routes in passerines. Therefore, we first review and clarify the definition of vagrancy and how it can arise from, but also differs from, migration and other types of movements. Second, we discuss potential proximate and ultimate mechanisms that may be responsible for the recent increase of YBWs in Europe in the light of proximate and ultimate mechanisms of migration and vagrancy. Third, we summarize the published information on the temporal and spatial occurrence of YBW in Europe. Fourth, we suggest possible empirical studies to distinguish between the migration and vagrancy hypotheses, and derive from that a road map to disentangle their respective contributions to a novel migration system in YBW. Finally, we interpret the current state of knowledge and assess why the understanding of this phenomenon should help us to gain important insights into the evolution and mechanisms of avian migration.

## Vagrancy or migration?

a) *Review of concepts*

Seasonal latitudinal migration is defined as a regular and recurring movement of birds between breeding and non-breeding areas [37–39]. Seasonal migration (hereafter, we simply refer to migration as defined above) is a population-level phenomenon, which concerns all individuals of a population or only a fraction of them (partial migration) but is part of their “normal” behaviour and is adaptive (or at least it is not selected against; see [40, 41] for comprehensive overviews on the genetics and evolution of avian migration). Many migratory species, especially migratory songbirds, rely on a genetic program inherited from their parents that will guide them during their inaugural migration [20, 42–45]. This endogenous migration program contains phenotypic components such as direction, duration and timing [31] that control the migratory behaviour and contribute to its variation [46, 47]. Moreover, migrants must have the innate ability to process intrinsic (e.g. body condition, [48]) and extrinsic (e.g. wind, [49]) cues as the ability of individuals to correctly respond to variation in such cues will determine the success of their migration [50–54].

Vagrancy is defined as the appearance of an individual outside the usual distribution range of its species (breeding, wintering or migrating range; see [10, 11]). Hence, identifying a vagrant is challenging as what constitutes the usual distribution of a species is itself hard to delimit precisely. For most species that undertake regular movements (dispersal, migration or prospection), abundance is not geographically uniform and it is impossible to draw a line between the area of usual distribution and the area where the species occurrence is rare enough to qualify as vagrancy (see also the “pseudo-vagrancy theory” in [29]). In their review, Lees and Gilroy [10] proposed the idea that the geographic range of a species should encompass something like 99.99% of individuals at a given time, and that anything outside this range might be defined as a vagrant.

Vagrancy can have intrinsic causes (e.g., the innate information about direction and duration of migration would not lead to the expected migratory destination of the population; [27, 28, 55–57]) and/or extrinsic causes (e.g. extreme weather, social adherence to a wrong species; [58, 59]). It can therefore result from excess of movements in the right direction (e.g. post-breeding dispersal, overshooting or longer exploration trips: [60]), or from an orientation of movements different from the species' typical patterns (see [10] for a detailed review). For long-distance migratory species, deviations from the optimal routes have usually dramatic consequences on fitness [61]. This is particularly the case for passerines because the migratory costs in terms of time and energy are very high [54, 62]. It is thus suspected that vagrants which fail to reorient likely die before reproduction [11, 55].

What would distinguish vagrancy from migration in general? Vagrancy is what migration is not: vagrancy is an individual-level phenomenon, it is not part of the normal behaviour of the species and, most importantly, it is generally not adaptive. For a vagrancy-like behaviour to become adaptive, it needs to be transmitted from one generation to the next, increase in frequency in the population, become the normal behaviour of a subset of the population and would thus form the basis for the evolution of seasonal migration along a novel route or other type of regular movement. The process could be gradual and over time could establish novel population-specific migration programs, including adaptations in morphology, physiology, and behaviour [31], which may be adapted to the local geographic environment where the population occurs (see Sect. 5).

Some authors only consider vagrancy as the natural variability of the migratory phenotype where inexperienced juveniles use a broad range of orientation at the start of their first migration which can be accentuated by exogenous factors [59, 63, 64]. Following this idea, distinguishing migration and vagrancy can be very challenging. Yet, the study of the vagrancy patterns showed that they were surprisingly stable, in most cases uncorrelated with the meteorological patterns and far from what we should expect from broad or random orientation (e.g. [10, 28, 65, 66]). For the YBW in Europe, the distinction between migration and vagrancy is straightforward because the common distribution of the species, i.e., Siberia in summer and Southeast Asia in winter, is geographically well separated from Europe ([24], see Fig. 1) so that the large numbers of YBWs arriving in Europe are either vagrants or seasonal migrants on a novel route but are not the outliers of a large distribution during migration or in winter.

b) *Potential proximate mechanisms of YBW vagrancy*

If vagrancy can have intrinsic and/or extrinsic causes, mechanisms responsible for the vagrancy behaviour are still poorly understood. They certainly depend on the movement that is exaggerated or wrongly expressed (e.g. migration, dispersal, feeding trip, exploratory journey) as well as on the traits and ecology of the species involved [28].

Since vagrancy is non-adaptive, it has no ultimate (selective) causes, in spite of the sometimes-proposed misconception that vagrancy could be favoured “for the good of the species”, as a way to promote colonization of new areas or adaptation to environmental changes. The unit of natural selection is generally the individual or the gene, not the group, population or species (except in rare cases, [67–70]): a trait or a behaviour can only be selected as long as it increases the survival or reproduction of the *individuals* that exhibit it, not if it is favourable to its *species*. Some hypotheses suggest that a small amount of variation in migration behaviour within the progeny of an individual can be favoured (e.g. the bet hedging hypothesis in [71]; or the scatter of the preferred migration in [72]). However, they do not predict the evolution of vagrancy in the YBW, where individuals go in a very different direction from the ancestral one as these hypotheses still require that most offspring would reach a favourable area and survive to reproduce. In these models, the advantages of the bet-hedging strategy are indeed to maximize, on average, the number of returning offspring of a reproducing individual. Any mechanism that decreases the fitness of an individual (e.g. by reducing the proportion of reproducing offspring) will be counter-selected. This makes vagrancy an interesting evolutionary puzzle that can only be resolved by understanding the proximate causes of this behaviour. While an extensive discussion of these mechanisms is beyond the scope of this paper, we provide here a few hypotheses concerning the YBW specifically.

For Siberian passerine vagrants including the YBW, it has been shown that meteorological factors alone do not explain their occurrence [28, 73]. On the contrary, Siberian birds appearing in Europe have been suggested to follow mirror-image [55, 65, 66] or reverse [28] migration route to their usual one, indicating that they use a different direction at the start of their migration [10, 27, 28]. This is indeed supported by recoveries within Europe (Fig. 3) and migratory orientation of YBW which have reached Europe and were tested in Emlen-funnels [27] or tracked by radio tags [57]. The causes of this misorientation are likely related to their endogenous program [27, 28, 55, 74] but so far neither mirror-image nor reverse migration offer a sole, complete satisfactory explanation for the vagrancy pattern of YBW in Europe [10]. The magnetic calibration hypothesis (tested on Pied Flycatcher’s nests, *Ficedula hypoleuca*; [75]) has been proposed as a possible proximate cause of the vagrancy phenomenon for Siberian species [11]. Again, it alone does not offer a complete satisfactory explanation because only some specific species express very strong vagrancy behavior among those breeding in the same Siberian areas. Other factors must thus come into consideration to explain why the abundance of vagrants of certain species has increased or varied over the past few decades. A westward expansion of the breeding range [8, 76] as well as an above-average breeding success [59] may explain why an increasing number of birds are observed in Europe. Interannual variations in abundance could also be linked to weather conditions (especially the wind) which may block or facilitate their arrival in Western Europe or by short variations of the Earth’s magnetic field caused by solar wind or flares [10, 77].

How can we explain that juvenile YBWs orient their migration towards Europe instead of South-eastern Asia, i.e., why do they show misorientation? Firstly, since migratory orientation in many passerines is a genetically encoded trait [20, 78], vagrants could have accumulated mutations in the gene(s) coding for orientation (hence there could be heritable differences between vagrants and normal migrants). However, this is probably unlikely to explain alone the substantial and currently well-established YBW vagrancy pattern as such a large number of individuals carrying strongly deleterious mutations would mean a high frequency of such mutations, which is hard to explain given the supposed strong selection against them.

Secondly, vagrancy could be entirely due to a different processing of orientation cues and their development during ontogeny, irreversibly affecting the migration phenotype without any change at the DNA level (i.e., phenotypic plasticity [46, 79]). Mirror-image and reverse migration are usually explained as incorrect use of inherited compasses: birds failed either to identify the reference point on the compass (i.e. north; reverse migration: [28]) or failed to use the correct angle with respect to the north–south reference line (mirror-image: [65]). Thorup [27] proposed that YBWs occurring in Europe (and likely originating from the western breeding populations) may have mirrored their natural migratory direction by 180°, taking the opposite initial great circle route direction from the ancestral migration route [80] (note that a mirror-image of their rhumb-line trajectory may also explain the occurrence of the species in southern Europe [10]). Underlying reasons for the different expressed compass orientation could be related to developmental issues during ontogeny [81] and compass integration [82], without genetic modifications.

Again, these two explanations are not mutually exclusive and vagrants could be a mix of genetically affected individuals and individuals whose orientation system has been modified by non-genetic mechanisms leading to increased variation and novel routes [46, 83].

## Temporal and spatial patterns of occurrence of YBW in Europe

**Fig. 2:**
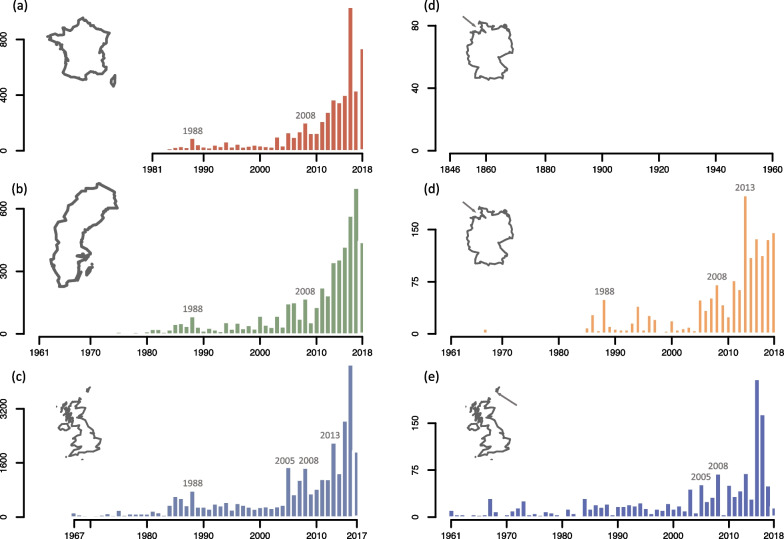
Total autumn numbers of Yellow-browed Warbler (*Phylloscopus inornatus*) in European countries: France (**a**), Sweden (**b**), the United Kingdom (**c**); and bird observatory: Helgoland, Germany (**d**), Fair Isle, Scotland (**e**). For Helgoland, the two panels span the 1846–2018 period (with a different scale). Years above bars indicate massive influxes recorded simultaneously in different locations. Data were collected from the literature or citizen-science database by the authors (for France: [87], https://www.faune-france.org/; Sweden: national reports published by BirdLife Sweden [128]; Helgoland: Jochen Dierschke; United-Kingdom: Scarce Migrant Committee, see [129]; Fair Isle: Fair Isle Bird Observatory)

The last few decades have seen a substantial rise in the number of recorded YBW in many countries in Europe (Fig. 2); most records have occurred after 1985 [84] with a number of publications additionally highlighting exceptionally high numbers of birds in so-called ‘influx’ years [85–87]. First observations dates range from 1845 (Germany [88]); 1937 (Norway [89]), to 1987 (Bulgaria [90]); 1988 (Poland [91]) and 2013 (Romania [84]). In several countries, the number of records has grown so much that it has lead the YBW to be classified as a regular migrant, rather than a rare migrant, or vagrant [84, 88], particularly in Great Britain. There it was removed from the British Birds Rarity Committee list in 1963 and after 2018 has no longer been reported in the ‘Scarce Birds Report’ having amassed annual totals of minimum 1950 in the preceding three years. Importantly, the increase in the number of records was noted independently of the increase in the number of observers in some locations (e.g. in Fair Isle, Scotland, and Helgoland, Germany, where the presence of long-established bird observatories with regular monitoring activities ensured that the increase was real and not due to increase in observation effort, Fig. 2).

Compare these figures to those recorded in other nations and it is clear that the distribution of records across the continent is far from even. De Juana [25] collated records from across annual rarities committee reports and from published papers, noting that Great Britain, Sweden, France and the Netherlands dominate the picture, with coastal areas contributing most. Differences in the observer effort notwithstanding, records of YBW in the Iberian Peninsula were under 1% of those recorded in United Kingdom, and under 8% of those in both Sweden and France, but even here a 2–3 fold increase has been documented in the last 10 years [92]. In Hungary, Romania and Bulgaria, which sit much closer to the Siberian breeding grounds of the YBW but considerably further south than the aforementioned countries, there are only a handful of records (Hungary: 6 [93]; Romania: 10 [94]; Bulgaria: 5 [95]), most of which come from ringing records, as opposed to observations.

Whilst the bias of records towards the northwest of the continent could be considered as partial proof of a reverse migration phenomenon (see [29] for discussion), the number of observers in United Kingdom, the Netherlands, Sweden, Norway and France, is far higher than in eastern European countries. This discrepancy probably explains the lower numbers of observations in the latter countries. Notably, the Richard’s Pipit, another Siberian regular migrant, was shown to stop-over during both spring and autumn migration in central European countries, but it is very rarely observed there [8], see also [92]. However, the north–south gradient in abundance of YBW in autumn across western Europe is real, as the number of birds seen per day in the field in autumn is much larger in coastal areas or islands of North-West Europe than in the Mediterranean area: one record in the Balearic Islands (Spain) against 220 in Fair Isle (Shetland, Scotland) in 2015 [96]. This geographic pattern in abundance is one of the puzzles of the occurrence of YBW in Europe as it does not fit with a scenario of random orientation from the breeding grounds. Note that some particular sites in the Mediterranean region, such as the island of Linosa (Italy), still differ from this pattern because in some years many YBW were recorded there [97]. However, it is currently difficult to know whether this results from an island isolation effect or from the existence of a more direct route to unknown wintering places on the African continent.

The vast majority of birds are recorded during the autumn, with Krüger & Dierschke [88] estimating a 46:1 ratio of autumn to spring birds and Illa et al. [92] reporting a 73:1 autumn to spring ratio in the Catalonian region of Spain. Interestingly, spring records have recently also increased, but the growth has been far slower, less linear and appears to have followed the rise in autumn records; for example, although autumn records in Belgium, the Netherlands and Germany began long before 1985, their spring records did not start until well into the 1990s. YBW in autumn are generally recorded from late September to early November although there is annual variation presumably due to weather patterns delaying or accelerating arrival. Peak arrival times in Scandinavia appear to precede those elsewhere in Europe, occurring in the last two weeks of September [25]; whereas the peak in Great Britain occurs often in the first two weeks of October, and the peak in France just slightly after this [25, 87, 98]*.* Although north-western European countries record just a few birds during the winter months (average per winter 1986–2021 in United-Kingdom: 8; the Netherlands: 4), Spain and Portugal regularly record birds all the way through November, December and January, with a recent high of 33 records on 1^st^ January 2015 in Portugal [98] and a record of 29 individuals during the winter of 2013–2014 in Lanzarote [85]. At the same latitude, the first cases of wintering of the species were noted in Morocco from 2018 onwards and also suspected in Mauritania [99]. In the Middle East, a few cases of wintering have also been noted during the last decade, but the situation is there more complex since the very similar Hume's Leaf Warbler (*Phylloscopus humei*) is also a regular winter visitor in this region and more abundant than in western Europe.

**Fig. 3:**
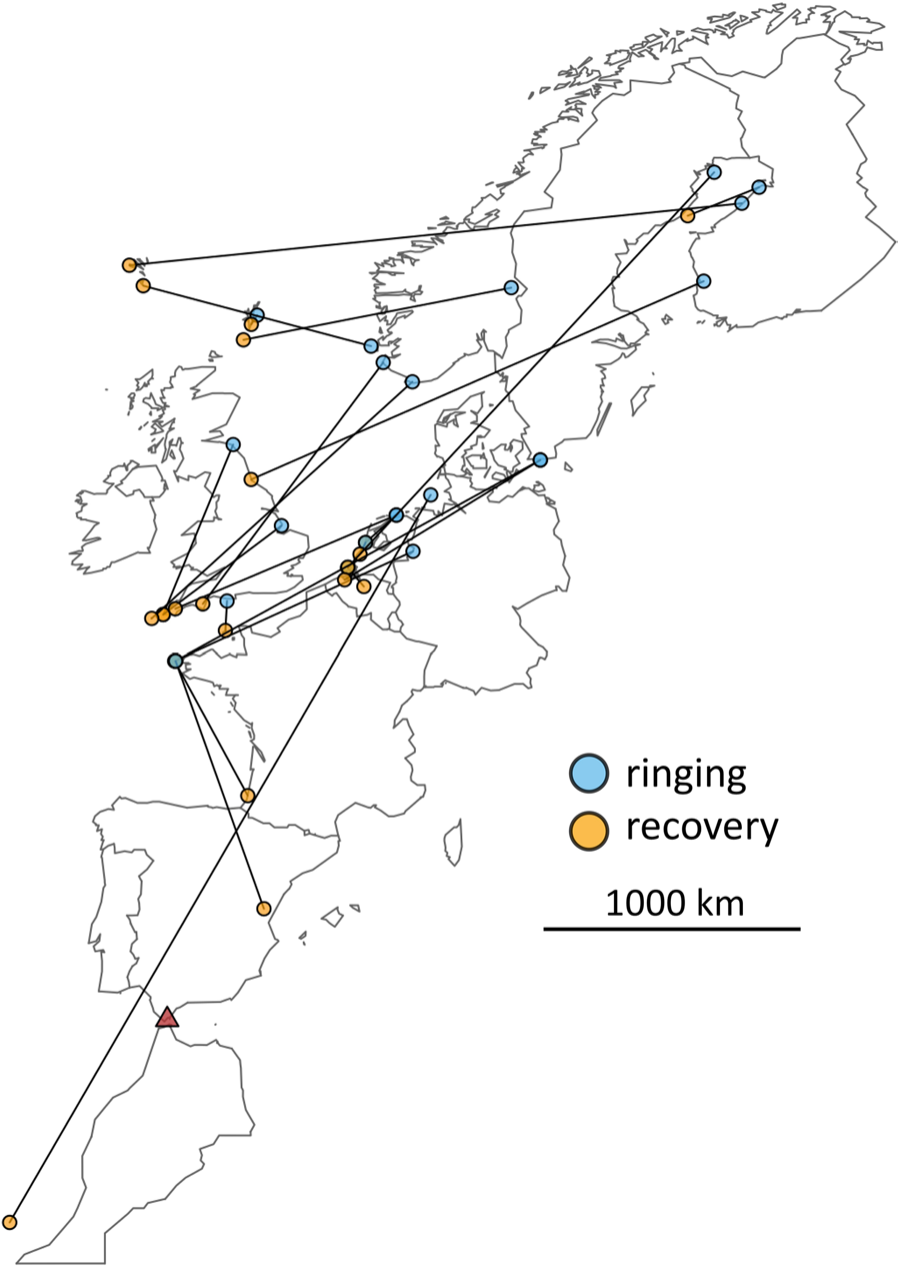
Recovery sites (orange) of 22 Yellow-browed Warblers (*Phylloscopus inornatus*), which were ringed in Europe during a same autumn season (blue; EURING data). The red triangle indicates the interannual and winter ringing recovery from Spain near Tarifa [26]

These timings appear to indicate a general direction of travel of moving individuals across the continent from Siberia in a westerly-south westerly direction overall [10], with some regional differences encountered. East–west orientated birds were found by Thorup [27] on the island of Christiansø in Denmark and highlighted by ringing activities (Fig. 3). A YBW captured on Helgoland in autumn 2013 was notably re-sighted in Lanzarote in January 2014, which demonstrated a southwest migration direction [85] (Fig. 3). Tonkin and Gonzalez-Perea [26] reported the recapture of a known, ringed individual in Andalucia, Spain over two consecutive winters, proofing that at least some YBWs have a high winter site fidelity. Although the few re-sightings provide only anecdotal evidence about the movement ecology of this species, they still fill important parts in our knowledge gaps about the movements of YBWs.

In addition to the observations in Europe, the YBW has also been recorded with increasing regularity along the western coast of North America in the last decades. Several YBWs were recorded in Alaska [100]. More recent records were made at even more southern latitudes with notably one in Baja California Sur in 2006 (Mexico, [101]), followed by two recent records in California and in British Columbia (Canada, [102]). These records probably reflect a global spread of the species which may also coincide with the increase in numbers in Europe.

## Road map to disentangle the respective contributions of vagrancy and migration

The recent increase in occurrence of YBWs in Europe provides a unique opportunity to study the underlying mechanisms of vagrancy and explore whether they could foster the evolution of a novel migration route in this species. Despite the fact that the species breeds in areas difficult to access and where the density of observers is relatively low, this small species has the advantage, unlike many other Siberian species, of being easy to catch, easy to detect, very suitable for caging, e.g. for orientation experiments, and well-known by bird observers. To assess whether YBW is establishing a new migration route towards Western Europe during autumn migration and to better understand the vagrancy approach, we propose different approaches in the Table 1.

**Table 1:** Proposals of empirical tests to disentangle the relative contribution of vagrancy and migration to the occurrence of the Yellow-browed Warbler (*Phylloscopus inornatus*) in Europe

Project	Context	Implications	Description	Challenges
Ageing YBWs across Europe	The low survival probability of young individuals (inferred by the small size of the YBW [103]) and the probable costs of reaching a region (northwestern Europe) whose climate is very different from the normal wintering habitats in Southeast Asia imply that most vagrants will probably not survive after the first outbound migration. On the contrary, since migrant passerines generally follow the same migratory route during their life [104, 105], individuals returning to the breeding area will head towards the previous wintering ground in subsequent years	If only juveniles in Europe: only compatible with vagrancy If adults and juveniles in Europe: compatible with both vagrancy and migration but a large proportion of adults would support the occurrence of regular migration to Europe	1) Ageing with plumage: adult birds undertake a complete post-breeding moult between July and September whereas juveniles undertake a partial post-juvenile moult between July and September involving head, body and probably some wing-coverts and occasionally some tail feathers [106]. Experienced ringers may therefore find a difference in structure and ground colour of greater-, median-, lesser-coverts, primaries and tail-feathers that can allow ageing 2) Proving the return of the same individuals in successive winters by marking individuals in wintering areas	1) Ageing this species remains challenging even for ringers with direct experience of the species, while most European ringers have no experience with adult YBWs 2) Evaluating age-ratio could be challenging because of a differential occurrence of adult and juvenile birds at coastal ringing sites [72, 107]: adults could use a more direct migration route through Central Europe. In addition, further efforts should be made to age birds that are observed early during autumn migration, especially in southern Europe and North Africa as adults might migrate before juveniles [108]
Orientations of YBWs in Europe	Birds that go west will end up in the Atlantic and will not be able to survive or re-orient to find suitable wintering conditions. Conversely, if some birds spend the winter in the Iberian Peninsula, they are expected to orient, at some point, south or southwest. Expectations are different depending on European regions	For localities in Western Europe (e.g., Norway, Shetlands, Western France): if only west, only compatible with vagrancy (in these locations) In same locations and elsewhere: if west to south, compatible with both vagrancy and migration	1) Orientation in modified Emlen-funnels for innate heading of migration direction (i.e., with access to only the Earth’s magnetic field, with access to the stars or a combination of cues; [109, 110]) 2) In free-flight conditions with the Motus network technology, considering external factors and heading direction (i.e., access to stars, wind/rain, ecological barriers ahead; [111]) 3) Route simulations based on known preferred orientation and alternative compass mechanisms [112–114]	External factors can influence the flight direction in field experiments
Inheritance of the westward orientation	Vagrancy may or may not have a genetic basis, but migration orientation is under genetic control in passerines	If no genetic inheritance of the westward orientation: only compatible with vagrancy If inherited genetic basis: compatible with both vagrancy and migration	1) Genomic analyses: by comparing the genome of individuals using westward and eastward orientation, we might be able to find genomic regions involved in the determination of orientation (e.g. Delmore et al. 2016, 2020) 2) Cross-breeding experiments: the heritability could be tested by reproducing individuals with known orientation and testing the orientation of their F1 offspring	Breeding small insectivores over several generations is highly challenging
Breeding origin of YBWs reaching Europe	Vagrant YBWs can come from anywhere within the breeding range of the species, but we expect that birds from the western part of the distribution are more likely to reach Europe and therefore will be overrepresented in Europe [27]	If YBWs seen in Europe come from different breeding localities: compatible with both vagrancy and migration If all YBWs breeding in a distinct section of the breeding range migrate to Europe: only compatible with migration	With a two-step process: 1) isotopic analyses from feathers collected in Europe would determine if birds that migrate west originate from only a “rather small” area or come from the entire breeding range [115]. See also [116] for a combined approach of several methods 2) if this step identifies a particular area where YBWs in Europe come from, field work in this area to catch breeding YBWs and sample their feathers grown during winter to check with isotopes if all birds migrate west in this area	The results of the isotope analyses will depend on the quality of sampling across the entire breeding range to calibrate the models
Tracking of YBWs reaching Europe	In contrast to vagrants, regular migrants are expected to return to the breeding grounds, transmit the information of the westward migration route to their offspring and return to Europe to winter	If some YBWs tracked from Europe return to the breeding ground and migrate back to Europe: compatible with, but not proof of, migration	Catch and deploy tracking device (i.e., light level geolocator: GLS) on YBWs in Europe in winter	GLS small enough for YBW are not yet available. Low site fidelity in winter would reduce retrieval rate
Population dynamics	Investigating changes in breeding distribution as well as understanding the impact of conditions that will influence breeding output of different breeding populations, success of migration from Siberia to Europe and survival of birds attempting to over-winter in Europe will help to determine the cause	If breeding success across a wide part of the range and conditions promoting successful migration to Europe are important: more compatible with vagrancy. If over-winter conditions in Europe are important for number next year: more compatible with migration	Relate numbers occurring in Europe each year to: weather conditions in breeding areas (will determine breeding success), weather conditions during autumn migration through Siberia, weather conditions in wintering areas in Europe. Ideally compare historical and current information on breeding range, breeding habitat, densities and reproduction success	Data availability: data to directly determine population size and breeding success in Siberia are unlikely to be available so it is necessary to use ecological proxies, which may not accurately capture causes of variation

## Interpretation of the current knowledge

**Fig. 4:**
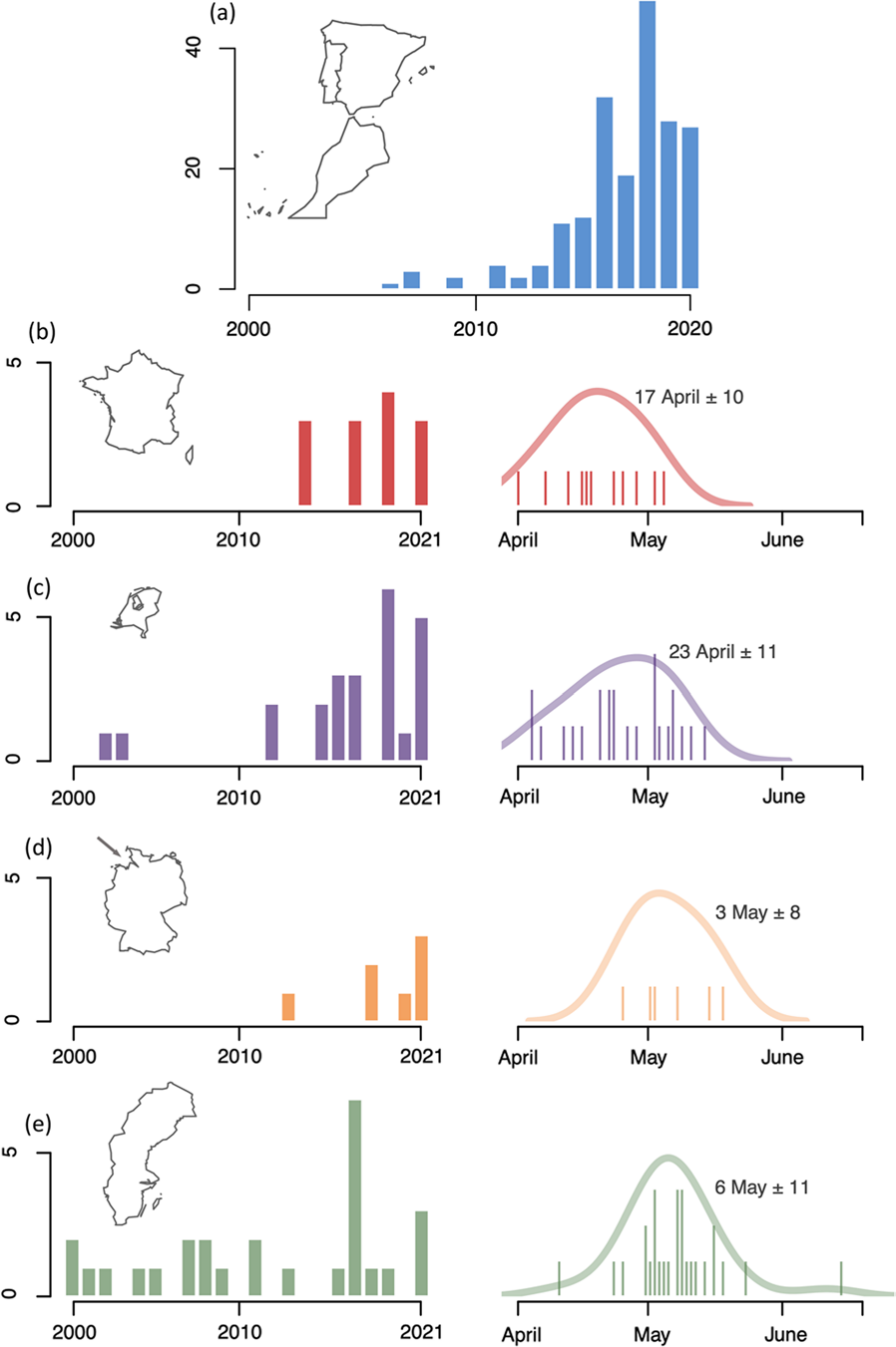
Number of wintering locations in Morocco, Spain and Portugal over the last two decades **a** and spring numbers of Yellow-browed Warbler (*Phylloscopus inornatus*) in several European countries: France **b**, the Netherlands **c**, Helgoland, Germany **d** and Sweden **e**. In a, numbers correspond to wintering locations noted in Morocco, Spain and Portugal, extracted from GBIF [130] for the months of December, January and February over the last two decades and thinned for each year for a distance of 5 kms. Numbers must be interpreted with caution due to change in observer effort over the last decades and reporting bias in citizen-science databases. For spring numbers, left panels indicate the number of spring records per year per country over the last two decades and right panels indicate the cumulated date records over the same years with the mean date of the passage. A density curve, estimated with the *density* function in R, highlights the phenology of the passage. For spring numbers, data were collected from citizen-science database by the authors (for France: https://www.faune-france.org/; the Netherlands: www.waarneming.nl; Helgoland: Jochen Dierschke; Sweden: https://www.artportalen.se/)

Although numbers of YBW have undoubtedly increased in Europe in recent decades (Fig. 2), several arguments suggest that they are still at least mainly composed of vagrants. Firstly, many (if not most) observations come from Western and North-Western Europe (e.g. Norway and British Isles), outside what we might assume to be a direct route from their breeding localities to southwestern Europe. These are probably birds flying westwards or following a reverse ‘great-circle’ route of their ancestral migration route [27, 57]. Ringing recoveries between Scandinavia and the Faroes or Shetlands (Fig. 3) and the occurrences of the species in Iceland (also rarely in the Azores; see sighting locations on Fig. 1) suggest that at least a few, and maybe many more, of the YBWs seen in Europe end up in the Atlantic Ocean [57]. Secondly, a large proportion of the birds observed in Europe appear to be juveniles that ended up in Europe in the course of their first migration. This is supported by age estimates at ringing stations. In Ottenby, Sweden, 100% of birds (*n* = 53) captured between 2010 and 2021 were identified as likely juveniles (M. Hellström unpublished data). In Rybachy, Russia, only one bird out of 45 (2%) captured between 1991 and 2013 showed a complete skull ossification and might thus have been an adult (N. Chernetsov unpublished data; see also [117]). Thirdly, the species remains extremely rare in Europe in spring, with numbers corresponding to one or two percent of those in autumn, depending on localities (see Fig. 4). Additionally, the recent increases in autumn pre-date the winter and spring increases and are therefore likely to be their cause, rather than a consequence of them. In summary, vagrancy seems to be by far the main cause of the YBW's occurrence in Europe.

Some recent evidence, however, suggest that a small fraction of these YBWs may be regular migrants that spend winter in southern Europe or northern Africa, return to their breeding grounds where they transmit their migratory orientation. In addition to the observations listed above, such as the status change from a rarity to a regular autumn migrant [87, 92], increasing numbers of overwintering YBWs in the Iberian Peninsula and North-Africa have been noted (Fig. 4; see [25, 26, 118]), as well as spring sightings at dates and phenology consistent with a return of birds to their breeding sites (Fig. 4; [24]). Furthermore, it is possible that spring migrants would use a more direct migration route through Central Europe during the pre-nuptial migration (proposed in [29] and showed for Richard's Pipit in spring and autumn in [8]). This would explain the lower ratio of records in spring than in autumn due to the possible lower detection probability along a continental route through Central Europe than along the coast (Fig. 1). Moreover, a substantial ringing effort carried out on the species in recent years has yielded ring recoveries indicating a re-orientation of birds towards the south-east after they reached the westernmost part of France in Brittany (see Fig. 3; birds ringed in Ouessant, France). These recoveries suggest that some birds could re-orient towards Iberian Peninsula or North-Africa once they reach the Atlantic coast (i.e. where they could find suitable wintering conditions [26]). Finally, a more detailed examination of birds captured in southwestern France suggests the presence of good candidates for adult birds among those captured during autumn [87]. Further studies are needed to demonstrate that this is a small but regular proportion of wintering birds in southwestern Europe and not a few exceptions. At this stage, we cannot ensure that these birds are migrants. Alternatively, they are either vagrants that survived their first year in Europe and took the same route the following year or adults that travelled along this route due to any potential proximate mechanisms detailed in Sect. 1.b.

Based on this information and on the ideas outlined in Table 1, it is clear that we need more evidence to assess the evolutionary status of YBWs occurring in Europe and the relative contribution of vagrancy and migration to the occurrence of the species in Europe. A true demonstration of the existence of a new migration route would require showing that a part of individuals that migrate to Europe in autumn survive, successfully breed and that their offspring also migrate to Europe before returning to the breeding area for reproduction (or that many individuals reproducing in a given area of the breeding range wintered in Europe, see Table 1). It should be then demonstrated that the birds using this new migration route form a self-sustaining population that is not entirely dependent on an influx of autumn vagrants to maintain it. This level of evidence seems difficult to reach in the near future but we can gain some insight from the other approaches outlined in the road map in Table 1.

## Perspectives in the study of avian migration and conclusion

We believe that the YBW offers a great opportunity for investigating evolutionary hypotheses deeply rooted in the classical literature of bird migration [119]. The species stands as an ideal model to study the potential emergence and selection of a migration route in the wild and also to investigate the determinism of vagrancy and orientation.

The large number of birds engaging in what it seems to be an evolutionary dead-end every autumn makes this phenomenon an interesting evolutionary puzzle to solve. A westward expansion of the breeding range associated with high reproductive success is likely be a part of the underlying process [76], as suggested by the increasing occurrence in Europe of species of Siberian origin known to have an expanding distribution (e.g., Red-flanked Bluetail *Tarsiger* *cyanurus* [120]). However, it would be surprising that it explains alone the large numbers of YBWs observed every autumn in Europe. Indeed, assuming a fixed percentage of the population ends up as vagrants in Europe, we should observe a similar trend of increasing numbers on ancestral migration routes in Asia. Considering the increases observed in some European countries (e.g. from dozens to thousands of individuals in two decades, see Fig. 1), the increases in Asia should have been similarly spectacular and detected simultaneously at several ringing stations in China or Mongolia. Hence, further studies of the orientation of YBWs within its breeding range should first provide more insight into variations in migration orientation towards its ancestral wintering areas. We also do not understand why this species is so abundant in Europe compared to the other Siberian species that regularly appear as vagrants in the west; estimates of population sizes for several breeding passerines in Siberia would be very useful in this context to assess if all species provide a similar proportion of vagrants or if YBW is peculiar in that respect. The increase in the number of YBW records outside Europe (e.g. western coast of North America) suggests that the YBW (or at least several of its population) is expanding demographically [59, 121].

If evidence arises that YBWs reaching Western Europe include regular migrants, this species would then also provide a model for understanding the evolutionary pathways of changes in migration orientation. While this is outside the scope of this paper, we wish to stress that this question is highly important, but is far from trivial. How the orientation of migration can change through selection will depend on the genetic architecture of orientation (from single-locus to highly polygenic determinism), on the mode of selection (directional versus disruptive) and on the geographic context (evolution in an isolated subpopulation or along a geographical gradient). One can easily understand how the orientation can change gradually in an isolated population with directional selection. For example, if an ancestral population is split into distinct glacial refugia, one of which has access to favorable wintering grounds that are closer to its breeding grounds than the ancestral wintering grounds and equally favorable. In theory, the shorter the migration distance to sites of equal quality, the higher the fitness, leading to gradual evolution of new migration routes. Accordingly, we mainly know of cases of migratory divide in secondary contact, when two populations joined after distinct glacial refugia [122–124]. Yet, rare cases of primary migratory divide also exist in birds [23].

In the case of the YBW and other Siberian species which could evolve a westward migration route from vagrants, the transition involves a phase where a very small number of individuals reproduce in a population exhibiting a radically different orientation phenotype. In a first case, special circumstances that promote reproductive isolation (and hence avoid panmixia) might favor the establishment of a new migration route, used by increasing numbers of individuals. Vagrant-like individuals should thus either come (and return) from isolated sub-populations areas where they should reproduce together or arrive sufficiently early/late compared to individuals using the regular route (phenologically mediated assortative mating as proposed in [23]). In a second case with no strong reproductive isolation, if the new migration orientation is encoded by a co-dominant allele at a single locus, most of their offspring will exhibit an intermediate migration phenotype. In this case, the fate of the new alleles would thus depend on the suitability of the wintering grounds and migration routes used by these individuals with intermediate orientation phenotypes. This is true of all evolutionary changes in migration orientation involving qualitative changes (such as the newly evolved northwestern orientation of central European Blackcap [20, 23], or westward migration of Richard’s Pipit [8]), yet this particular issue of the fate of the first pioneers has remained largely unaddressed. Theoretical work possibly involving simulations would help understanding the conditions under which vagrants can give birth to new migration routes. In that perspective, 19th century literature indicates that the YBW may have been a regular autumn visitor in Europe during the nineteenth century [125, 131]. On Helgoland (Germany), the species has been recorded since 1845 and almost annually in autumn until 1890 (Fig. 2; Gätke also noted the near-absence of the species in spring [125]). Even if it is puzzling that there are no records of this species in other European countries for that time, this pattern could reflect a possible historical dynamic in the occurrence of this species in Europe (the same is true for Richard's Pipit; see [8, 125]). The relative high number of YBWs records on Helgoland and its absence in England at the same time is intriguing and raises questions about the existence of new migration routes and the temporal consistency of these.

## Conclusion

In this paper, we presented the YBW as a model to understand the mechanisms of vagrancy and its potential significance for the evolution of new migratory routes. While vagrancy has long fascinated researchers and layman alike, several issues have been discussed in the literature and interpreted differently. We wish that the present review will stimulate the discussion about vagrancy and whether or not vagrants may truly act as pioneers in the discovery of new migratory routes.

We also wish to stress that understanding the occurrence of the YBW in Europe will rely on the collaboration of numerous research teams, birdwatchers, ornithologists, ringing stations across Europe, North-Africa and Asia and we hope that this project will participate in building a strong network of collaborators, which could also provide help for launching other projects in the future.

## Data Availability

Sources of the data used to produce the figures are provided in the text. Authors can be directly contacted for further development and questions about the data used to produce the figures.
