# Correction: The Yellow-browed Warbler (*Phylloscopus inornatus*) as a model to understand vagrancy and its potential for the evolution of new migration routes

**DOI:** 10.1186/s40462-023-00368-3

**Published:** 2023-01-31

**Authors:** Paul Dufour, Susanne Åkesson, Magnus Hellström, Chris Hewson, Sander Lagerveld, Lucy Mitchell, Nikita Chernetsov, Heiko Schmaljohann, Pierre‑André Crochet

**Affiliations:** 1 LECA, CNRS, Univ. Grenoble Alpes, Univ. Savoie Mont Blanc, Grenoble, France; 2 Department of Biological and Environmental Sciences, University of Gothenburg, Gothenburg, Sweden; 3 Gothenburg Global Biodiversity Centre, Gothenburg, Sweden; 4 Department of Biology, Center for Animal Movement Research, Lund University, Ecology Building, 22362 Lund, Sweden; 5 Ottenby Bird Observatory, Öland, Sweden; 6 British Trust for Ornithology, The Nunnery, Thetford, Norfolk, IP27 2PU UK; 7 Wageningen University & Research, Ankerpark 27, 1781 AG Den Helder, Netherlands; 8 Environmental Research Institute, Centre for Energy and Environment (CfEE), The North Highland College UHI, Ormlie Road, Thurso, KW14 7EE UK; 9 Ornithology Lab, Zoological Institute RAS, 1 Universitetskaya Emb, 199034 St. Petersburg, Russia; 10 Department of Vertebrate Zoology, St. Petersburg State University, 7‑9 Universitetskaya Emb, 199034 St. Petersburg, Russia; 11 Institute for Biology and Environmental Sciences (IBU), Car Von Ossietzky University of Oldenburg, Carl‑Von‑Ossietzky‑Straße 9‑11, 26129 Oldenburg, Germany; 12 Institute of Avian Research, An Der Vogelwarte 21, 26386 Wilhelmshaven, Germany; 13 CEFE, CNRS, Univ Montpellier, EPHE, IRD, Montpellier, France

**Correction to: Movement Ecology (2022) 10:59**

**https://doi.org/10.1186/s40462-022-00345-2**

Following publication of the original article [1], the authors identified missing data in panel d of Fig. 2 due to a typesetting mistake. The correct Fig. 2 is included in this Correction and the original article has been corrected.

**Fig. 2:**
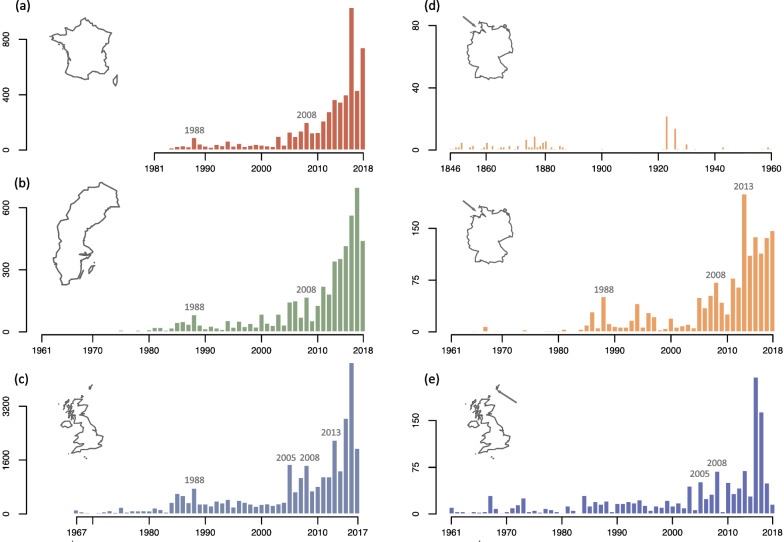
Total autumn numbers of Yellow-browed Warbler (*Phylloscopus inornatus*) in European countries: France (**a**), Sweden (**b**), the United Kingdom (**c**); and bird observatory: Helgoland, Germany (**d**), Fair Isle, Scotland (**e**). For Helgoland, the two panels span the 1846–2018 period (with a different scale). Years above bars indicate massive influxes recorded simultaneously in different locations. Data were collected from the literature or citizen-science database by the authors (for France: [87], https://www.faune-france.org/; Sweden: national reports published by BirdLife Sweden [128]; Helgoland: Jochen Dierschke; United-Kingdom: Scarce Migrant Committee, see [129]; Fair Isle: Fair Isle Bird Observatory)

The publisher apologises to the authors and readers for the inconvenience caused by the error.

## References

[1] Dufour (2022). The Yellow-browed Warbler (*Phylloscopus inornatus*) as a model to understand vagrancy and its potential for the evolution of new migration routes. Mov Ecol.

